# The Effect of Muscle Strength on Marathon Race-Induced Muscle Soreness

**DOI:** 10.3390/ijerph182111258

**Published:** 2021-10-27

**Authors:** Marilia Santos Andrade, Carolina Ribeiro Lopes Ferrer, Rodrigo Luiz Vancini, Pantelis Theodoros Nikolaidis, Beat Knechtle, Thomas Rosemann, André Luis Lacerda Bachi, Aldo Seffrin, Claudio Andre Barbosa de Lira

**Affiliations:** 1 Department of Physiology, Federal University of Sao Paulo, São Paulo 04021-001, Brazil; marilia1707@gmail.com (M.S.A.); carolinarlferrer@gmail.com (C.R.L.F.); netoseffrin@gmail.com (A.S.); 2 Center of Physical Education and Sports, Federal University of Espirito Santo, Vitória 29075-910, Brazil; rodrigoluizvancini@gmail.com; 3 School of Health and Caring Sciences, University of West Attica, 11521 Athens, Greece; pademil@hotmail.com; 4 Medbase St. Gallen Am Vadianplatz, Vadianstrasse 26, 9001 St. Gallen, Switzerland; 5 Institute of Primary Care, University of Zurich, 8091 Zurich, Switzerland; thomas.rosemann@usz.ch; 6 Department of Otorhinolaryngology, Federal University of São Paulo, São Paulo 04021-001, Brazil; allbachi77@gmail.com; 7 Post-Graduation Program in Health Sciences, Santo Amaro University (UNISA), São Paulo 04829-300, Brazil; 8 Human and Exercise Physiology Division, Faculty of Physical Education and Dance, Federal University of Goiás, Goiânia 74690-900, Brazil; andre.claudio@gmail.com

**Keywords:** DOMS, endurance, isokinetic strength, pain, peak toque, runners

## Abstract

Background: Muscle soreness after a competition or a training session has been a concern of runners due to its harmful effect on performance. It is not known if stronger individuals present a lower level of muscle soreness after a strenuous physical effort. The aim of this study was to investigate whether the pre-race muscle strength or the $\dot{V}O_{2}max$ level can predict muscle soreness 24, 48 and 72 h after a full marathon in men. Methods: Thirty-one marathon runners participated in this study (age, 40.8 ± 8.8 years old; weight, 74.3 ± 10.4 kg; height, 174.2 ± 7.6 cm; maximum oxygen uptake, $\dot{V}O_{2}max$, 57.7 ± 6.8 mL/kg/min). The isokinetic strength test for thigh muscles and the $\dot{V}O_{2}max$ level was performed 15–30 days before the marathon and the participants were evaluated for the subjective feeling of soreness before, 24, 48 and 72 h after the marathon. Results: The participants presented more pain 24 h after the race (median = 3, IQR = 1) than before it (median = 0, IQR = 0) (*p* < 0.001), and the strength values for the knee extensor muscles were significantly associated with muscle soreness assessed 24 h after the race (*p* = 0.028), but not 48 (*p* = 0.990) or 72 h (*p* = 0.416) after the race. The $\dot{V}O_{2}max$ level was not associated with the muscle pain level at any moment after the marathon. Conclusions: Marathon runners who presented higher muscular strength for the knee extensor muscles presented lower muscle soreness 24 h after the race, but not after 48 h or 72 h after the race. Therefore, the muscle soreness level 3 days after a marathon race does not depend on muscle strength.

## 1. Introduction

Marathon races are among the most popular mass sporting events worldwide, with over 2.1 million participants around the world annually [1]. The 42,195 m of a marathon race represent an impressive effort imposed onto the human body, and it is well-documented that unusual or exhaustive exercise, whether mechanical or metabolic in nature, can cause exercise-induced muscle damage [2,3]. Indeed, muscle soreness is frequently reported by athletes after a marathon race [2,3,4]. Moreover, loss of muscle strength, muscle length and range of motion have also been reported after exhaustive exercises [3,5]. These transitory conditions can negatively affect the running economy [6], increase the blood lactate concentration [7], and reduce the maximum oxygen uptake ($\dot{V}O_{2}max$) [8,9]. Considering that there is a consensus that the performance in long-distance events depends on the physiological factors cited above (running economy, $\dot{V}O_{2}max$ and the sustained percentage of $\dot{V}O_{2}max$) [10], it is reasonable to assume that the presence of muscle soreness can compromise athletic performance in training sessions in addition to delaying the return to training after a marathon race.

Despite the fact that complete pathophysiology of muscle soreness after exhaustive exercise is not completely understood, several possible mechanisms have been proposed [11]. The most accepted hypothesis is that the pain results from muscle microinjuries caused by eccentric movement [12]. After that, there are lysis in the sarcolemma and release of cytolytic enzymes and myoglobins, causing inflammation, edemas and production of free radicals [12,13]. This inflammation is characterized as an enzymatic reaction with inflammatory mediation, with elements such as thromboxanes, prostaglandins and leukotrienes coming from cyclooxygenases and lipoxygenases that cause increased vascular permeability and sensitization of nerve fibers, mainly of types III and IV [14].

Many strategies have been studied to treat or even prevent muscle soreness [14,15,16,17,18,19,20,21]; however, it is unknown if muscular strength or $\dot{V}O_{2}max$ values are associated with muscle soreness after exercise. This knowledge could be useful for athletes and coaches in creating strategies that allow the runner to return to normal training as quickly as possible, without prejudice to the performance caused by pain [22].

Therefore, the aim of this study was to investigate whether pre-race muscle strength and $\dot{V}O_{2}max$ can predict muscle soreness 24, 48 and 72 h after a full marathon in men. We hypothesized that the stronger athletes would present with less muscle soreness in the days following a marathon race, and we also hypothesized that $\dot{V}O_{2}max$ would present no association with muscle soreness following a marathon race.

## 2. Materials and Methods

### 2.1. Participants

Thirty-five male amateur runners who applied for the 2017 São Paulo Marathon (Brazil) participated in the study. The invitation was made by the race administration.

The inclusion criteria to participate in the study were to be enrolled in the 2017 São Paulo Marathon with at least two years of running practice. The exclusion criteria included having acute pain in the lower limbs, edemas, taking medicines which affect the musculoskeletal system or not finishing the race. Of the 35 athletes who replied to the initial invitation, four athletes did not finish the race and were excluded from the study. Therefore, thirty-one athletes participated in the entire study.

The athletes were 40.8 ± 8.8 years old, weighted 74.3 ± 10.4 kg and were 174.2 ± 7.6 cm tall, had 8.2 ± 8.0 years of running experience and the distance covered per week was 48.7 ± 24.6 km. The subjects were informed of the benefits and risks of the investigation prior to signing an institutionally approved informed consent document to participate in the study. All the experimental procedures met the ethical standards of sports and exercise science research [23] and were approved by the Human Research Ethics Committee of the University Cruzeiro do Sul and the National Research Ethics Committee (CONEP) under number CAAE: 67318317.3.0000.8084 on 17 April 2017. The study conformed to the principles outlined in the Declaration of Helsinki [24].

### 2.2. Procedures

Each participant reported to the laboratory two days in the same week, with an interval of 2 or 3 days to prevent the results of one test from being influenced by the results of the other [25,26]. Moreover, the athletes were instructed to avoid training within 24 h before each visit to the laboratory.

On the first visit, they answered a questionnaire about training habits; afterwards, anthropometric data measurements and the cardiorespiratory maximum text were performed. On the second visit, the isokinetic strength test was performed. The two visits were performed 15–30 days before the marathon. The race times were provided by the organizing committee of the race.

Before the marathon and 24, 48 and 72 h after the marathon, the athletes were asked about the subjective feeling of soreness. To avoid the invasive nature of muscle biopsies and plasma creatine kinase activity to assess muscle damage, muscle soreness was assessed using a pain scale. Pain Likert scales were also used in previous studies to assess the perceived soreness [5,27].

### 2.3. Questionnaire about Training Habits

The athletes answered a questionnaire about training habits with the following two open-ended questions: (1) How many years have you been practicing running?; (2) How many kilometers do you run a week?

### 2.4. Isokinetic Strength Test

All the participants underwent a knee extensor muscles isokinetic evaluation for the dominant lower limb. Isokinetic strength tests were performed on a Biodex System 3 isokinetic dynamometer (Biodex Medical System, Shirley, New York, NY, USA). A calibration procedure was performed prior to each test. The dominant lower limb was determined by asking the participant which limb they prefer to use to kick a ball. This procedure was used in previous studies [28,29].

Before the strength test, the participants warmed up for five minutes on a cycle ergometer (Cybex Inc., Ronkonkoma, NY, USA). This cycle ergometer was set at a resistance of 25 Watts [29]. The warm-up was followed by low-intensity dynamic stretching exercises for the lower limbs [30].

Then, the participants were asked to sit with their hips flexed at approximately 85°. Initially, the participants underwent three trials at submaximum effort with a gradually increasing load and then performed one set of five repetitions at the maximum concentric contraction at angular speeds of 60°/s in the concentric mode. A coefficient of variance lower than 10% was considered valid for analysis. Standardized verbal encouragement was given to the participants during the entire test, always by the same experienced examiner.

The peak torque in newton-meters (Nm) was assessed at 60°/s for the knee extensor muscles at concentric action and recorded for further analysis.

### 2.5. Cardiorespiratory Maximum Test on a Treadmill

Cardiopulmonary exercise testing (CPET) was conducted on a motorized treadmill (Inbrasport, ATL, Porto Alegre, Brazil) using a computer-based metabolic analyzer (Quark, Cosmed, Italy). The calibration procedure was performed prior to each test. CPET was used to measure $\dot{V}O_{2}max$. $\dot{V}O_{2}max$ was determined as stabilization of $\dot{V}O_{2}$ (increase lower than 2.1 mL/min/kg) even after increasing the treadmill velocity during the last stage of the CPET [31]. All the participants reached the $\dot{V}O_{2}max$ values.

The test protocol consisted of a warm-up phase of 3 min at 9 km/h. After the warm-up period, the running velocity was increased by 1 km/h every minute until voluntary exhaustion [32]. The entire CPET lasted for 8–12 min and the treadmill grade was set at 1% to simulate the energy cost of outdoor running [33].

### 2.6. Perceived Soreness

Muscle soreness was assessed using a seven-point Likert scale (Table 1) for muscle pain individually immediately before and remotely (using Google Forms) 24, 48 and 72 h after the race. A study collaborator would call the participant to remind him to answer the questionnaire after the race.

### 2.7. Statistical Analysis

The strength, $\dot{V}O_{2}max$ and race time values presented normal distribution and homogeneous variance according to the Shapiro–Wilk and Levene’s tests, respectively. An ordinal logistic regression model was fitted to describe and explain the relationship between the peak knee extensor torque value at 60°/s and $\dot{V}O_{2}max$ with the pain scale 24, 48 and 72 h after the race. The Akaike information criterion (AIC) identifies the model quality, but its meaningfulness depends on there being a good predictive model [34]. Statistical analysis was performed using SPSS v21.0 (Chicago, IL, USA) [35]. The significance level was set at *p* < 5%. The data are presented as the means ± standard deviation for the following data: $\dot{V}O_{2}max$, race time, and the peak torque values, as well as the median and the interquartile range for pain values.

## 3. Results

$\dot{V}O_{2}max$ and the total marathon race time in the group were 57.7 ± 6.8 mL/min/kg and 4:09:36 ± 0:42:36 (data presented as h:mm:ss), respectively.

**Figure 1:**
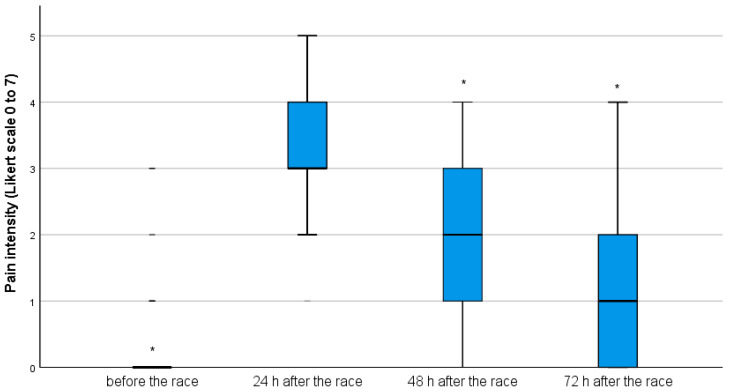
Boxplot for pain intensity before the race and 24, 48 and 72 h after the race (* *p* < 0.05—lower pain level than 24 h after the race).

Before the race, the median and the interquartile range for the pain level reported by the participants (0(0)) were significantly lower than the values reported 24 h after the race (3(1)) (*p* < 0.001). The pain level reported 48 h after the race (2(2)) was significantly lower than 24 h after the race (*p* < 0.001). Similarly, the pain level reported 72 h after the race (1(1)) was also lower than 48 and 24 h after the race (*p* < 0.001); therefore, the peak of pain was reached 24 h after the race for the entire group (Figure 1).

The level of association between the knee extensor strength and $\dot{V}O_{2}max$ with the pain level reported 24, 48 and 72 h after the marathon race were also studied. The pain level 24 h after the marathon presented a significant association with the knee extensor muscles strength (x^2^ (1) = 4.821, *p* = 0.028, ß = −0.023), with a reduction of 0.023 points on the pain scale (IC = −0.004/−0.002) per unit of increment in the peak extensor muscles torque at 60°/s. Conversely, there was no association with the $\dot{V}O_{2}max$ level (x^2^ (1) = 0.242, *p* = 0.623, ß = −0.025). For this model, the AIC was 96.780. On the other hand, the pain level 48 h after the marathon presented no association with the knee extensor muscles strength (x^2^ (1) = 1.128, *p* = 0.288, ß = −0.010) and $\dot{V}O_{2}max$ (x^2^ (1) = 0.003, *p* = 0.958, ß = 0.003), and the AIC was 97.768. Similarly, the pain level 72 h after the marathon also presented no association with the knee extensor muscles strength (x^2^ (1) = 0.662, *p* = 0.416, ß = −0.008) and $\dot{V}O_{2}max$ (x^2^ (1) = 0.013, *p* = 0.905, ß = −0.006), and the AIC was 98.096.

## 4. Discussion

This study intended to investigate whether the pre-race muscle strength can predict muscle soreness 24, 48 and 72 h after a full marathon in men. The main findings of the study are that the knee extensor muscles strength was significantly associated with muscle soreness 24 h after the marathon race (the more muscle strength, the less muscle soreness), but this association was not present 48 and 72 h after the race. These findings confirm the initial hypothesis that the stronger athletes would present with less pain for at least 24 h after the race. The knowledge that muscle strength is associated with muscle soreness 24 h after the race indicates that stronger individuals recover faster from pain after the race, but does not indicate that they would be able to return to training faster. Previous research showed many mitochondria, erythrocytes, leukocytes and other phagocytic cells within the extracellular and extravascular spaces besides dilation and disruption of the T-tubule system after a marathon race, evidencing a process of muscle fiber necrosis and inflammation, which are most prevalent at 1 and 3 days after a marathon [36,37]. Nonetheless even after this period and after muscle soreness has subsided, there is some level of cellular damage in muscles as late as 8 weeks after a race [36,38]. Considering this muscle recovery time after a marathon, it is understood that the return to sports practicing after the race should not be immediate; however, the research regarding the return to running following a marathon is scarce and there is no consensus about whether rest is indicated [37]. Sherman et al. (1984) compared two groups of marathoners for 6 days following a race [39]. One group was subjected to rest and the other—to running exercise of gradually increasing duration. The authors showed that the resting recovery group had a slightly better muscle function recovery than the running group. On the other hand, in a very recent study, Martinez-Navarro et al. (2021) compared the effects of two exercise modalities (running or elliptical training) vs. rest on the time course of muscle damage recovery during the week after a marathon race [37]. The authors showed that the return to running 48 h post-marathon does not seem to have a negative impact on muscle damage recovery. It is evident that even in the most recent study that shows that returning to racing can be not harmful to muscle recovery, such as return does not occur before 48 h. The results of this study show that stronger individuals have less pain 24 h after a marathon, but this does not indicate that these individuals can return to training early because 48 h after a marathon, there is no association between strength and pain.

It is important to note that the runners who completed the São Paulo Marathon race presented with a significantly higher muscle pain 24 h after the run than before it. Nonetheless, the pain reduced significantly between 24 and 72 h after the race: twenty-four hours after the race, 45% of the runners experienced mild pain when walking on a flat surface and soreness, but 72 h after the race, only 9.7% of the runners had the same pain level. These findings are in accordance with the results of Tojima et al. (2016) and Tokinoya et al. (2020) who also found a significant increase in pain levels after a full marathon race, which peaked at 24 h after the race [2,40].

An important factor to be considered that could contribute to this high percentage of pain after the race is that the São Paulo International Marathon is a race that features a high altitude (378 m) and elevation loss (321 m), which is more than the most important marathons that take place around the world. For example, the elevation loss during the New York Marathon is 289 m, the Santiago Marathon—252 m, the Berlin Marathon—79 m, the Chicago marathon—73 m. The elevation loss is important because during downhill running, the eccentric action is of fundamental importance to support the body weight against gravity [41]. The knee extensor muscles are very important to absorb shock and avoid knee flexion and the consequent fall of the runner. Therefore, the knee extensor muscles action is accentuated during downhill running [42]. In this context, it is possible that stronger thigh muscles are less needed during downhill running and during the eccentric action, considering that the race pace is the same between the groups, and thus should suffer less mechanical stress in muscles and connective tissues resulting in less muscle damage and pain. It is worth mentioning that this is a hypothesis based on the findings; however, future studies are needed to test this hypothesis.

Other factors, such as the physical fitness level, may also contribute to muscle damage in response to intense exercise [43,44,45]. However, the results showed no association between the $\dot{V}O_{2}max$ values and the pain level after the race in none of the three moments. Despite this, future studies should be carried out to verify the effect of other variables on muscle soreness, such as the muscle typology.

In this study, muscle strength was measured in the concentric mode. However, considering that muscle damage occurs mainly after exercises presenting eccentric action, knowledge about the impact of the maximum eccentric strength of the thigh muscles on muscle soreness after a marathon would also be of great interest. The authors suggest that future studies be carried out to elucidate this issue.

A strength of the study is related to the experimental protocol. Muscle soreness was studied after a real full marathon race, which increases the ecological validity of the study. Several previous studies investigated muscle soreness by submitting the participants to downhill running under experimental conditions [21,46,47,48]; however, it is not common for runners to perform training sessions with these characteristics.

A limitation of this study is that muscle soreness was assessed using a pain scale in which an athlete would answer questions about the delayed pain that the runner was feeling, but no muscle biopsies or plasma creatine kinase activity to assess the presence of muscle damage was included; therefore, it is not possible to affirm that the referred pain after exercise was really due to delayed-onset muscle soreness (DOMS); it is possible that a feeling of fatigue could also have been evaluated by the runners.

## 5. Conclusions

The results of this study suggest that the muscle strength level of the knee extensor muscles can predict muscle pain 24 h after a marathon race, but not after 48 and 72 h. Moreover, the $\dot{V}O_{2}max$ level cannot predict muscle soreness 24, 48 or 72 h after a full marathon race.

## Figures and Tables

**Table 1:** Muscle pain assessment scale used in the study.

Value	Description
0	Painless
1	Mild pain when touching/vague pain
2	Moderate pain when touching/small persistent pain
3	Mild pain when walking up or up the stairs
4	Mild pain when walking on a flat surface/sore
5	Moderate pain, stiffness or weakness when walking on a flat surface/very sore
6	Severe pain that makes movement difficult

## Data Availability

The data presented in this study are available on request from the corresponding author. The data are not publicly available due to privacy and ethical reasons.
